# Bragg–Williams Theory for Particles with a Size-Modulating Internal Degree of Freedom

**DOI:** 10.3390/molecules28135060

**Published:** 2023-06-28

**Authors:** Guilherme Volpe Bossa, Sylvio May

**Affiliations:** 1 Instituto de Ciencias Físicas y Matemáticas, Universidad Austral de Chile, Valdivia 5110566, Chile; 2 Department of Physics, North Dakota State University, Fargo, ND 58108-6050, USA; sylvio.may@ndsu.edu

**Keywords:** phase transition, free energy, molecular reorientation, isotherm, common-tangent construction

## Abstract

The field of soft matter teems with molecules and aggregates of molecules that have internal size-modulating degrees of freedom. Proteins, peptides, microgels, polymers, micelles, and even some colloids can exist in multiple—often just two dominating—states with different effective sizes, where size can refer to the volume or to the cross-sectional area for particles residing on surfaces. The size-dependence of their accessible states renders the behavior of these particles pressure-sensitive. The Bragg–Williams model is among the most simple mean-field methods to translate the presence of inter-particle interactions into an approximate phase diagram. Here, we extend the Bragg–Williams model to account for the presence of particles that are immersed in a solvent and exist in two distinct states, one occupying a smaller and the other one a larger size. The basis of the extension is a lattice–sublattice approximation that we use to host the two size-differing states. Our model includes particle–solvent interactions that act as an effective surface tension between particles and solvent and are ignorant of the state in which the particles reside. We analyze how the energetic preference of the particles for one or the other state affects the phase diagrams. The possibility of a single phase-two phases-single phase sequence of phase transitions as a function of increasing temperature is demonstrated.

## 1. Introduction

The level of detail for modeling the phase behavior of particle systems ranges from atomistic descriptions [1,2,3,4] to simple lattice representations that are treated on the level of the mean-field theory [5,6,7]. Each limit offers advantages and challenges: the former is able to account for all chemical details and interactions but may be too demanding in terms of what is computationally feasible and may not always offer physical interpretations for what is being observed. The latter affords a straightforward calculation of approximate phase diagrams that can be understood in every detail, yet is subject to simplifications—often drastic ones—in molecular detail and in the accuracy of the phase diagram. The present work focuses on the latter case and asks how a model that is simplified as much as possible (a lattice representation using mean-field theory) may account for the presence of an internal degree of freedom that affects the effective spatial extension of the phase-separating particles.

Among the systems that have their properties affected by internal degrees of freedom are hydrogels [8,9], elongated and globular proteins [10,11,12], polymers [5,13,14], and mechanosensitive channels that contribute to the regulation of transport through cellular membranes [15,16,17]. Microgels, more specifically, are polymeric gel particles [18] that possess the ability to adjust their volume in response to external factors, such as pH, temperature, and ionic concentration [19,20,21]. Compared to regular-sized hydrogels, the relative volume changes of microgels are larger and occur faster [20,22]. Another example of a system where the effective size of molecules plays a role is that of Langmuir monolayers. A Langmuir monolayer is a single-molecule-thick layer formed by surface active/amphiphilic molecules at the air–water interface [23,24,25]. Langmuir monolayers have served as a model system for studying, for example, the interaction of lipids with cholesterol [26,27], DNA adsorption onto thin films [28,29], and the interactions of bioactive molecules [30,31] and antimicrobial peptides with bacterial membranes [32,33]. A change in the effective size of a molecule in a Langmuir monolayer can be accomplished by a simple molecular reorientation. This renders the adopted molecular orientations pressure-sensitive [34]. The analysis of isotherms—the relationship how the monolayer area varies upon changing the lateral pressure at constant temperature—provides indirect information about the type of molecular assembly and phase behavior. However, isotherms do not directly reveal information about molecular orientations and interactions between molecules. Theory and simulation can help to create links, and, indeed, theoretical and computational [3,35] approaches have been proposed to rationalize the role played by inter-molecular interactions in systems where molecules are able to reorient. This is the case, for example, for colloids trapped at a dielectric interface [6,36,37].

On the level of mean-field theory, we have previously presented [7] a lattice–sublattice model that accounts for two molecular orientations of particles trapped at the air–water interface. Although this model provides reasonable interpretations for experimentally obtained surface pressure–area isotherms [38,39,40,41], it suffers from neglecting inter-molecular interactions. Here, we present an extension of this model where inter-molecular interactions are accounted for on the mean-field level [5,42]. More specifically, we consider a system composed of particles that are immersed in a solvent and that exist in two distinct states, one occupying a smaller and the other a larger size. Our model applies equally to two-dimensional and three-dimensional systems. We assume that particle–solvent interactions are present that act as an effective surface tension, independently of the state in which a particle resides. We focus our analysis on phase diagrams, which allows us to readily assess how the energetic preference of the particles for one or the other state affects the distribution of states and composition of coexisting phases.

## 2. Theory

**Figure 1:**
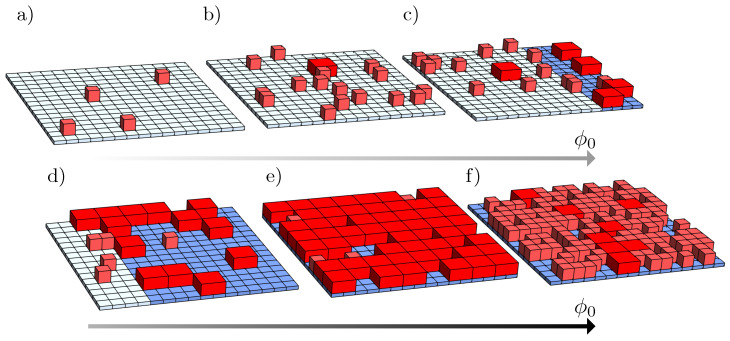
Schematic representation of a system composed of molecules present in two states: small (*S*, light red cubes) and large (*L*, dark red cuboids). From diagrams (**a**–**f**): illustration of how the system may phase-separate upon increasing $\phi_{0}$. The phases, here colored in white and blue, can assume different sizes and compositions depending on the values of $\phi_{0}$, λ, χ, and ξ. The gray arrows below the diagrams indicate the increase in $\phi_{0}$, with darker gray for larger $\phi_{0}$. Results illustrate a two-dimensional lattice for ξ=4.

We consider a binary mixture of solvent molecules and a fixed number of $N_{0}$ particles. The particles can reside in two distinct states with different particle volumes: a smaller volume $\nu_{0}$ and a larger volume $\xi \nu_{0}$, with ξ≥1. Throughout this paper we use the index “*S*” and “*L*” to denote quantities related to particles in the small-volume and in the large-volume state, respectively. The numbers of particles in each state, $N_{S}$ and $N_{L}$, add up to the fixed total number $N_{0}=N_{S}+N_{L}$. Assuming the particles are residing on a lattice of *M* equal sites, it is convenient to introduce the corresponding volume fractions $\phi_{L}=\xi N_{L}/M$ and $\phi_{S}=N_{S}/M$. Hence, the scaled number of particles $\phi_{0}=N_{0}/M=\phi_{L}/\xi +\phi_{S}$ is a fixed constant [7]; while $N_{S}+\xi N_{L}$ lattice sites are occupied by the $N_{0}$ particles, the remaining $M-N_{S}-\xi N_{L}$ sites are filled with solvent molecules. Here, we assume an *S* particle and a solvent molecule occupy one single lattice site each. More general cases with different volumes for solvent molecules and *S* particles are straightforward to implement. Figure 1 shows a schematic representation of a two-dimensional system of a square lattice composed of particles that can assume two distinct states: small (*S*, light red cubes) and large (*L*, dark red cuboids, with ξ=4). In the figure, we qualitatively illustrate a typical scenario of how the system may evolve upon increasing $\phi_{0}$: from a single phase composed of *S* particles—diagrams (a) and (b)—to two coexisting phases, colored in white and blue, and each with distinct compositions and phase size—diagrams (c) and (d). By further increasing $\phi_{0}$, as in diagram (e), the system exhibits a single phase composed mostly of *L* particles. Finally, for even larger $\phi_{0}$, diagram (f) displays the lattice densely packed with *S* particles. The color gradient in the arrows indicates the increase in $\phi_{0}$, with darker colors corresponding to larger values of $\phi_{0}$. The study of how different choices of interaction parameters bring about this and similar phase behaviors is one of the main goals of this work.

The Helmholtz free energy of the system, $F=-TS+U_{LS}+U_{int}$, is composed of an entropic contribution, −TS; energy terms related to switching particles between their *L*-state and *S*-state, $U_{LS}$; and interactions of particles with solvent molecules, $U_{int}$. The absolute temperature is denoted by *T* and the configurational entropy by S. A lattice–sublattice approximation proposed by Han and co-workers [43] allows us to estimate S as

(1) $$\begin{array}{c} \frac{-S}{Mk_{B}}=\frac{\phi_{L}}{\xi }\ln\phi_{L}+\left(\frac{1}{\xi }-1\right)\left(1-\phi_{L}\right)\ln\left(1-\phi_{L}\right)+\left(1-\phi_{L}-\phi_{S}\right)\ln\left(1-\phi_{L}-\phi_{S}\right)+\phi_{S}\ln\phi_{S} \end{array}$$

where $k_{B}$ denotes the Boltzmann constant. The energy contribution $U_{LS}$ is a single-particle energy that can be written as $U_{LS}/\left(k_{B}T\right)=\lambda N_{S}$, where λ quantifies the energy penalty required for a single molecule to switch from the *L*-state to the *S*-state. Finally, the particle–solvent interactions, per lattice site, are accounted for on the mean-field level by the expression

(2) $$\frac{U_{int}}{Mk_{B}T}=\chi \left(1-\phi_{L}-\phi_{S}\right)\left(\phi_{L}+\phi_{S}\right),$$

where χ characterizes the interaction strength between a lattice site occupied by a solvent molecule and a lattice site occupied by a particle. Our definition of the particle–solvent interaction effectively amounts to introducing a tension between the particles and the solvent. Positive tension favors not only the aggregation of particles but also the *S*-state over the *L*-state of isolated particles that are surrounded by solvent. Note that both λ and χ are dimensionless. For the sake of mathematical simplicity, Equation (2) is based on the assumption that solvent molecules interact with particles in the *S*-state and *L*-state in the same way, and *S* particles and *L* particles do not interact with each other. In a more general approach, one would assign distinct interaction strengths to pairs of *L* particles and solvent, *S* particles and solvent, and *L* particles and *S* particles. With the energy contributions specified above, the (dimensionless) free energy per lattice site, $f=F/(Mk_{B}T)$, reads

(3) $$\begin{array}{ccc} f(\phi_{L},\phi_{S}) & = & \lambda \phi_{S}+\frac{\phi_{L}}{\xi }\ln\phi_{L}+\left(\frac{1}{\xi }-1\right)\left(1-\phi_{L}\right)\ln\left(1-\phi_{L}\right)\\ & + & \left(1-\phi_{L}-\phi_{S}\right)\ln\left(1-\phi_{L}-\phi_{S}\right)+\phi_{S}\ln\phi_{S}+\chi \left(1-\phi_{L}-\phi_{S}\right)\left(\phi_{L}+\phi_{S}\right). \end{array}$$

To analyze how the interaction strengths λ and χ affect the number of particles residing in their *S*-state and *L*-state, we must first obtain the equilibrium distributions. To this end, we insert the relation $\phi_{L}=\xi \left(\phi_{0}-\phi_{S}\right)$ into Equation (3) and find the global minimum of $f(\xi \left(\phi_{0}-\phi_{S}\right),\phi_{S})$ as a function of $\phi_{S}$. That is, we solve

(4) $$\frac{\partial f(\xi \left(\phi_{0}-\phi_{S}\right),\phi_{S})}{\partial \phi_{S}}=0$$

for $\phi_{S}$, and from all solutions we select that of the lowest energy to obtain the optimal (equilibrium) distribution $\phi_{S}=\phi_{S}^{opt}\left(\phi_{0}\right)$ for fixed $\phi_{0}$ and any set of parameters ξ, λ, χ. Hence, the optimal value of the free energy, minimized for the number of *L* particles versus *S* particles, reads

(5) $$f\left(\phi_{0}\right)=f\left(\xi \left(\phi_{0}-\phi_{S}^{opt}\left(\phi_{0}\right)\right),\phi_{S}^{opt}\left(\phi_{0}\right)\right).$$

Known $f(\phi_{0})$ affords the calculation of phase separation via the familiar common-tangent construction [44].

## 3. Results and Discussion

**Figure 2:**
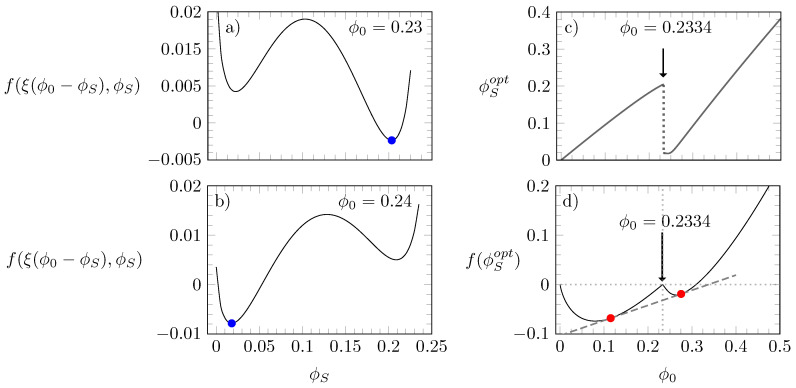
Diagrams (**a**,**b**): free energy $f(\xi \left(\phi_{0}-\phi_{S}\right),\phi_{S})$ according to Equation (3), calculated as a function of $\phi_{S}$ for $\phi_{0}=0.23$ and $\phi_{0}=0.24$; blue bullets mark the global minimum. Diagram (**c**): $\phi_{S}^{opt}$ as a function of $\phi_{0}$. Diagram (**d**): $f(\phi_{0})$ as a function of $\phi_{0}$ with red bullets marking the points that connect through a common tangent. In all four diagrams, ξ=4, λ=1.5, and χ=1.2.

We start our discussion by presenting in Figure 2 a series of diagrams that illustrate how the free energy and optimal distributions of states are calculated for a given set of λ, χ, and ξ; in this particular case, we chose λ=1.5, χ=1.2, and ξ=4. As mentioned above, the optimal value $\phi_{S}=\phi_{S}^{opt}$ is determined such that the free energy $f(\xi \left(\phi_{0}-\phi_{S}\right),\phi_{S})$ adopts a global minimum. Diagrams (a) and (b) of Figure 2 display $f(\xi \left(\phi_{0}-\phi_{S}\right),\phi_{S})$ as a function of $\phi_{S}$ for $\phi_{0}=0.23$ and $\phi_{0}=0.24$, respectively, with the global minimum being marked by a blue bullet in each case.

Upon increasing $\phi_{0}$ from 0.23 to 0.24, the position of the global minimum moves from $\phi_{S}\approx 0.2$ to $\phi_{S}\approx 0.02$. Diagram (c) reveals that the distribution of states changes discontinuously at $\phi_{0}=0.2334$. At this value, the function $f(\xi \left(\phi_{0}-\phi_{S}\right),\phi_{S})$ has two minima with equal depth. Furthermore, as shown in diagram (d) of Figure 2, if we calculate the free energy $f(\phi_{0})$ as a function of $\phi_{0}$, we observe that $\phi_{0}=0.2334$ is the value where $f(\phi_{0})$ adopts a local “cusp-like” maximum, thus necessitating the existence of a common tangent and, hence, two coexisting phases. Indeed, the red bullet diagram (d) in Figure 2 mark the points that exhibit a common tangent (dashed gray line); these correspond to the compositions of the two coexisting phases; each phase with its own value of $\phi_{S}^{opt}$.

**Figure 3:**
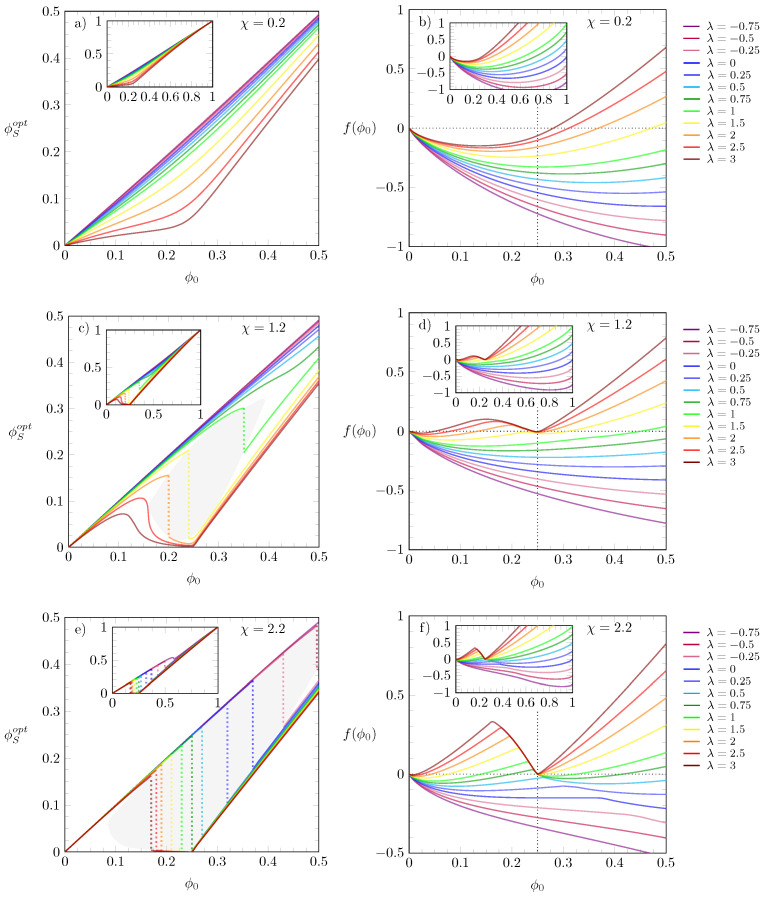
Left and right diagrams display the values of $\phi_{S}^{opt}$ and $f(\phi_{0})$, respectively, versus $\phi_{0}$ for different λ and fixed ξ=4. Each row corresponds to a specific value of χ: χ=0.2 (top), χ=1.2 (middle), and χ=2.2 (bottom). In all diagrams, the values of λ are color-coded according to the side legend on the right. The $\phi_{S}^{opt}$ values are solutions of Equation (4), i.e., the value of $\phi_{S}$ that globally minimizes the free energy $f(\xi \left(\phi_{0}-\phi_{S}\right),\phi_{S})$. The shaded gray areas mark the region where the $\phi_{S}^{opt}$ values change discontinuously, and the color-matching dotted lines indicate discontinuity of $\phi_{S}^{opt}$. Insets display the results over the entire range $0\leq \phi_{0}\leq 1$.

The results shown in Figure 2 correspond to a specific choice of the interaction parameters λ and χ. In order to account for other choices, we present in Figure 3 results for $\phi_{S}^{opt}$ and $f(\phi_{0})$ versus $\phi_{0}$ for λ values ranging from λ=−0.75 to λ=3 and three different choices of χ: χ=0.2 in diagrams (a) and (b), χ=1.2 in diagrams (c) and (d), and χ=2.2 in diagrams (e) and (f). Recall that $\phi_{S}^{opt}$ is the solution of Equation (4), thus being the value at which $f(\xi \left(\phi_{0}-\phi_{S}\right),\phi_{S})$ adopts a *global* minimum. The main diagrams magnify the region $0\leq \phi_{0}\leq 0.5$, whereas the insets display the results in the range $0\leq \phi_{0}\leq 1$; all results in Figure 3 are obtained for ξ=4, and the λ values are color-coded according to the legends on the side-panels. In diagrams (a) and (b)—top row—we observe that, irrespective of the values of λ, all distributions of $\phi_{S}^{opt}$ are continuous, and the free energies $f(\phi_{0})$ exhibit a single minimum. For $\phi_{S}^{opt}$, increasing λ produces results that evolve from straight lines (purple and blueish curves) to distributions that tend to bend at $\phi_{0}\approx 0.25$ (red and brown curves). For larger χ, as in panels (c) and (d), where χ=1.2, the $\phi_{S}^{opt}$-curves do not only bend but, for some values of λ, start to display a discontinuity analogous to that observed in Figure 2c. A similar behavior is also present in the bottom row of diagrams, but, as in this case χ is even larger (χ=2.2), the curves are discontinuous over a wider ranger of λ values; in both rows of diagrams we gray-shaded the area where the initial growth of $\phi_{S}^{opt}$ is followed by a discontinuous jump to smaller values, and color-matching dotted lines are plotted as a visual guide for this change. For larger χ, the free energy $f(\phi_{0})$ displays two minima, which become more pronounced upon increasing λ; for example, for χ=2.2, the larger the values of λ, the closer the minima to $\phi_{0}=0$ and to $\phi_{0}=1/\xi =0.25$.

The limit of small $\phi_{0}$ offers a transparent discussion of the different roles that λ and χ play for the particles to reside in the *S*-state or *L*-state; recall that λ is the energy cost required to switch a single particle from its large (*L*) to its small (*S*) size [7] and that χ accounts for the effective surface tension (a line tension in the two-dimensional case) that acts on the particles when in contact with the solvent.

Upon increasing $\phi_{0}$, starting from $\phi_{0}=0$, the lattice becomes populated with particles either in the *L*-state or *S*-state. When χ is sufficiently large, the surface tension between the particles and the solvent results in the *S*-state dominating. Growing $\phi_{0}$ gives rise to an increasingly large number of particle–particle interactions that replace particle–solvent interactions. For λ>0, where the *L*-state is energetically preferred over the *S*-state, this may lead to a transition to a dense phase dominated by the *L*-state. Let us analyze which state, *L* or *S*, dominates in the limit of small $\phi_{0}$. To this end, it is useful to calculate the slope of $\phi_{S}^{opt}$ as a function of $\phi_{0}$ for fixed λ and χ. A linear relation $\phi_{S}^{opt}∼\phi_{0}$ is valid in the limit of small $\phi_{0}$. Assuming $\phi_{S}^{opt}=s\phi_{0}$ in Equation (3) and expanding around $\phi_{0}=0$, we arrive at the following solution for the slope *s*:

(6) $$s=\frac{d\phi_{S}^{opt}}{d\phi_{0}}{|}_{\phi_{0}=0}=\frac{1}{1+\frac{1}{\xi }e^{-\chi (\xi -1)+\lambda }}.$$

**Figure 4:**
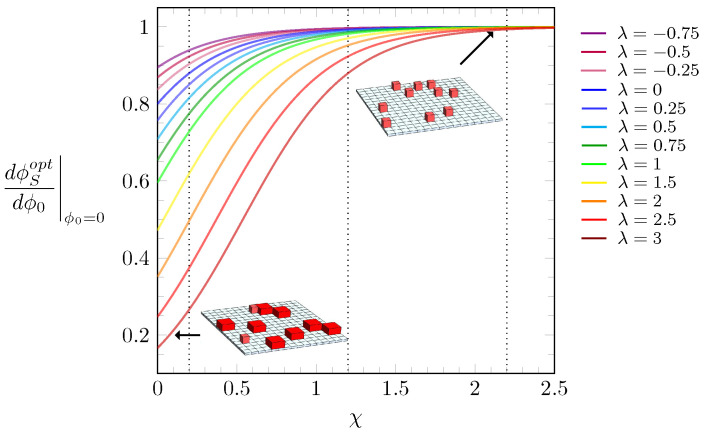
Slope from the free energy expansion around $\phi_{0}=0$ as a function of the interaction parameter χ, calculated according to Equation (6). The values of λ are color-coded according to the side legend. The black dotted lines mark the χ values used in the diagrams of Figure 3, i.e., χ=0.2, 1.2, and 2.2. The insets display illustrations of two systems, one with particles mostly in the *L*-state (bottom left) and another with all particles in the *S*-state (top right).

Results from Equation (6) are plotted in Figure 4 for different values of λ, color-coded according to the legend. To visualize the predictions of Equation (6), Figure 4 also displays system illustrations for small slopes, where most of the particles are in the *L*-state (bottom left inset), and another, for large *s*, where virtually all particles are in the *S*-state (top right inset). We note that the larger χ, the more particles reside initially in the *S*-state. Furthermore, if λ is also large, the values of $\phi_{S}^{opt}$ tend to be virtually zero right after the discontinuous jump of $\phi_{S}^{opt}$, as can be seen in orange, red, and brown curves in Figure 3e. Thus, any particle further added to the lattice will readily assume the *L*-state. This trend, however, does not continue when $\phi_{0}$ grows further: for large $\phi_{0}$, more particles in the *L*-state are placed on the lattice until steric constraints force them to undergo a transition to the smaller *S*-state.

**Figure 5:**
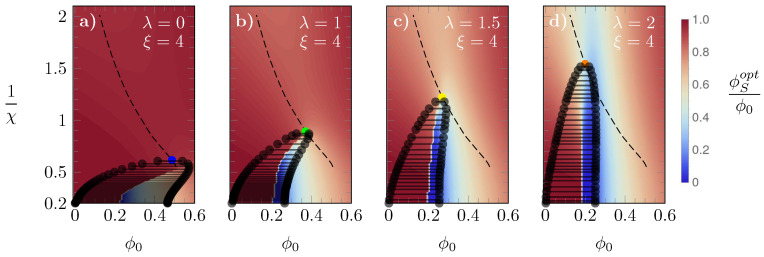
Phase diagrams, 1/χ versus $\phi_{0}$, for ξ=4 and different values of λ: λ=0, 1, 1.5, and 2 in panels (**a**–**d**), respectively. The critical value of χ is marked by colored bullets: blue (λ=0), green (λ=1), yellow (λ=1.5), and green (λ=2). The dashed line shown in all diagrams is the line on which all critical χ values lie. The “heat maps” in the background display the value of $\phi_{S}^{opt}/\phi_{0}$ color-coded according to the side legend.

In Figure 5, we display phase diagrams for ξ=4 and a different value of λ for each panel: λ=0 (a), λ=1 (b), λ=1.5 (c), and λ=2 (d); as it is common [44,45,46], we present our results in diagrams 1/χ versus $\phi_{0}$. Note that 1/χ is proportional to the absolute temperature *T* because χ is an interaction energy scaled by the thermal energy unit $k_{B}T$. In addition to displaying selected tie lines connecting the coexisting phases, the heat map in each panel shows the fraction $\phi_{S}^{opt}/\phi_{0}$, with values colored according to the sidebar legend. As we argue in our discussion of Figure 2, changes in $\phi_{S}^{opt}$ have a direct effect on how the free energies $f(\phi_{0})$ vary as a function of $\phi_{0}$. Specifically, discontinuous changes of $\phi_{S}^{opt}$ always imply the presence of a common tangent and thus a phase transition. This is indeed the case in the presented phase diagrams. The critical point, that is, the smallest value of χ at which for a given λ a phase transition can occur can be calculated [46,47,48] from solving the three equations

(7) $$\frac{\partial f(\xi \left(\phi_{0}-\phi_{S}\right),\phi_{S})}{\partial \phi_{S}}=0,\;\frac{\partial^{2}f\left(\xi \left(\phi_{0}-\phi_{S}\right),\phi_{S}\right)}{\partial \phi_{0}^{2}}=0,\;\frac{\partial^{3}f\left(\xi \left(\phi_{0}-\phi_{S}\right),\phi_{S}\right)}{\partial \phi_{0}^{3}}=0.$$

As our parameter space has five dimensions ($\chi ,\;\phi_{0},\;\phi_{S},\;\lambda ,\;\xi$), we can use Equation (7) to calculate three critical parameters (χ, $\phi_{0}$, $\phi_{S}$) in terms of the other two fixed variables (λ, ξ). The critical χ=χ(λ,ξ), here denoted by $\chi^{*}$, is marked by bullets in Figure 5, colored according to the same scheme as in the previous figures. The dashed line—reproduced in all diagrams of Figure 5—is the line on which all the critical points lie as λ is varied. Upon increasing λ, the critical $\chi^{*}$ and the corresponding $\phi_{0}$ are both reduced. Similarly to what was observed in Figure 2, this occurs because the larger the energy cost λ, the less favorable is the L→S switch. Furthermore, we also observe an evolution of the spinodal line towards a more uniformly convex and symmetric profile. Interestingly, the presence of a concave region of the spinodal line for small λ suggests the possibility of a single phase-two phases-single phase sequence of phase transitions. In diagram (a) of Figure 5, for example, decreasing the temperature (which corresponds to increasing χ) at fixed $\phi_{0}=0.5$ passes from a single phase to a two-phase region and, subsequently, back to a single phase.

**Figure 6:**
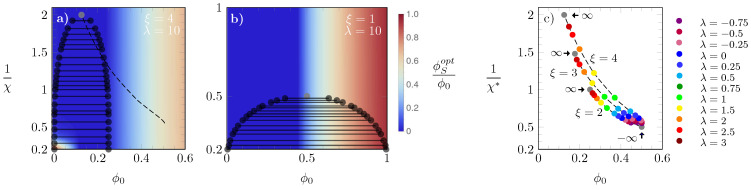
Phase diagrams for λ=10 with ξ=4 (**a**) and ξ=1 (**b**). The dashed line in (**a**) is the line along which the critical values of χ lie; the heat maps in (**a**) and (**b**) display the value of $\phi_{S}^{opt}/\phi_{0}$, color-coded according to the side legend in (**b**). Diagram (**c**): location of critical points, where each set of dashed lines and symbols correspond to ξ=2, 3, 4, from bottom to top lines; values of λ are color-coded as specified in the side panel, and the gray bullets mark the limits λ→±∞. For λ→−∞, all critical points merge into one location at $\chi^{*}=2$ and $\phi_{0}=0.5$, irrespective of ξ.

In order to investigate the effects of further increasing λ, in diagrams (a) and (b) of Figure 6 we show results obtained for λ=10 and two different choices of ξ: ξ=4 in (a) and ξ=1 in (b). The latter corresponds to the *S*-state and *L*-state having the same size, which leads to the same phase diagram as the regular Bragg–Williams free energy. Hence, in diagram (b), the critical point is $\chi^{*}=2$, $\phi_{0}=0.5$ and $\phi_{S}^{opt}=0.5$, whereas for diagram (a), the location of the critical point is close to $\chi^{*}=0.5$, $\phi_{0}=0.125$ and $\phi_{S}^{opt}\approx 0$. More generally, in the limit of large λ, where the *L*-state is enforced, the critical point is located at χ=2/ξ and $\phi_{0}=1/\left(2\xi \right)$. To bridge between these limiting cases, in panel (c) of Figure 6 we display how the critical point $\chi^{*}$ and corresponding $\phi_{0}$ vary as a function of $\phi_{0}$ for different choices of λ (symbols are color-coded according to the side legend).

The three sets of dashed lines, on which the corresponding symbols lie, were calculated for ξ=2, 3, and 4, from bottom to top. For each ξ, the gray bullets mark the results obtained in the limit of λ→−∞ (bottom right) and λ→∞ (left bullets, as indicated by arrows). In the limit of λ→−∞, the *S*-state is enforced. Hence, as in this case the system consists exclusively of particles in the *S*-state, all lines converge to the Bragg–Williams result irrespective of the value of ξ. In the opposite limit, i.e., λ→∞, all particles adopt the *L*-state. Consequently, the system behaves as composed solely of particles in the *L*-state, with particle volume $\nu =\xi \nu_{0}$, and we obtain the familiar Bragg–Williams result, but for a lattice with ξ times larger lattice sites. As λ=10 is close to the limit λ→∞, the mechanism above provides a rationale for the findings in diagrams (a) and (b).

We finally note that the function $f(\phi_{0})$ can be used to calculate [7] the pressure *P* of the system through

(8) $$v_{0}P=\phi_{0}\frac{df(\phi_{0})}{d\phi_{0}}-f\left(\phi_{0}\right).$$

**Figure 7:**
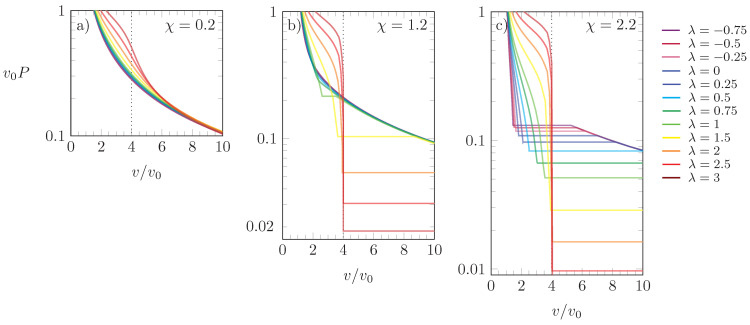
Scaled pressure $\nu_{0}P$ versus $\nu /\nu_{0}=1/\phi_{0}$ for χ=0.2 (**a**), χ=1.2 (**b**), and χ=2.2 (**c**). Different colors correspond to different values of λ, as indicated in the side legend. The dotted line marks the point $\nu /\nu_{0}=\xi$. Results calculated for ξ=4.

In Figure 7, we present isotherms for the scaled pressure $\nu_{0}P$ as a function of $\nu /\nu_{0}=1/\phi_{0}$ for three different choices of χ: χ=0.2 (a), χ=1.2 (b), and χ=2.2 (c). Different values of λ are color-coded according to the side legend, and all results were obtained for ξ=4. Because χ>0 corresponds to effectively attractive interactions between the particles, increasing χ always reduces *P* at fixed λ. Despite this, increasing λ at fixed χ does not always increase *P*, despite the growing preference for the *L*-state. The pressure tends to increase with λ for $\chi <\chi^{*}$ and for $\chi >\chi^{*}$ if $\nu <\nu_{0}$. However, for $\chi >\chi^{*}$ and $\nu >\nu_{0}$, increasing λ lowers the pressure of a phase-separated system because a larger degree of aggregation is induced that involves a smaller number of particles and, thus, a lower pressure.

## 4. Conclusions and Outlook

Molecules with internal degrees of freedom are ubiquitous in nature. Colloids, proteins, and microgels, are just a few examples of molecules that can adjust their size in response to external stimuli. In the present work, we developed a simple extension of the Bragg–Williams model to describe solvent-immersed particles that can exist in two states, one of a larger (*L*) and another of a smaller size (*S*). Our model is based on a lattice–sublattice approximation [7,43] and accounts for particle–solvent interactions on the mean-field level. More specifically, we considered two energy parameters: λ, the cost of a particle to switch from the *L*-state to the *S*-state, and χ, an effective particle–solvent interaction that acts analogously to a surface tension and is independent of the state in which the particles reside. We focused our analysis on how these parameters affect thermodynamic properties: the distribution of particle states as expressed by $\phi_{S}^{opt}$ and the occurrence of phase separation. Phase diagrams show how the energetic preference of the particles for one or the other state affects phase transitions and compositions of coexisting phases. We also calculated critical points and considered the effect of varying ξ, the size ratio between the particles in their *L*-state and *S*-state. In the limit of very large positive or negative λ values, our model recovers familiar results of the Bragg–Williams model. We designed our model to apply to systems composed of particles that can reside in two states of different size. However, its generalization to particles with more than two size-dependent states is straightforward. Other possible extensions account for interactions between particles in different states as well as distinct interactions between solvent molecules and particles in different states. Finally, our model has the capacity to serve as a tool to rationalize different contributions to experimentally observed “shoulders” in surface pressure–area isotherms [30,39,40] and phase transitions of monolayers [49,50].

## Data Availability

Not applicable.
